# Functional craniology and brain evolution: from paleontology to biomedicine

**DOI:** 10.3389/fnana.2014.00019

**Published:** 2014-04-02

**Authors:** Emiliano Bruner, José Manuel de la Cuétara, Michael Masters, Hideki Amano, Naomichi Ogihara

**Affiliations:** ^1^Centro Nacional de Investigación sobre la Evolución HumanaBurgos, Spain; ^2^Universidad Autónoma de MadridMadrid, Spain; ^3^Montana Tech, ButteMT, USA; ^4^Keio UniversityYokohama, Japan

**Keywords:** paleoneurology, cranial integration, brain shape, myopia, Alzheimer’s disease, thermoregulation, morphometrics

## Abstract

Anatomical systems are organized through a network of structural and functional relationships among their elements. This network of relationships is the result of evolution, it represents the actual target of selection, and it generates the set of rules orienting and constraining the morphogenetic processes. Understanding the relationship among cranial and cerebral components is necessary to investigate the factors that have influenced and characterized our neuroanatomy, and possible drawbacks associated with the evolution of large brains. The study of the spatial relationships between skull and brain in the human genus has direct relevance in cranial surgery. Geometrical modeling can provide functional perspectives in evolution and brain physiology, like in simulations to investigate metabolic heat production and dissipation in the endocranial form. Analysis of the evolutionary constraints between facial and neural blocks can provide new information on visual impairment. The study of brain form variation in fossil humans can supply a different perspective for interpreting the processes behind neurodegeneration and Alzheimer’s disease. Following these examples, it is apparent that paleontology and biomedicine can exchange relevant information and contribute at the same time to the development of robust evolutionary hypotheses on brain evolution, while offering more comprehensive biological perspectives with regard to the interpretation of pathological processes.

## INTRODUCTION

Since the beginning of morphometrics and evolutionary anatomy, students like Thomas Henry Huxley, D’Arcy Wentworth Thompson, and many others, suggested that some morphological characters are correlated during evolution by means of common factors or reciprocal influences and constraints. Olson and Miller published their book on morphological integration in 1958, and Moss and Young proposed their functional craniology in 1960, stressing the intimate structural relationships between brain and braincase (Olson and Miller, 1958; Moss and Young, 1960). Nonetheless, the analysis of anatomical integration requires multivariate statistics, large datasets, and powerful visualization tools, which have been provided only at the end of the 20th century, most of all by means of landmark-based approaches and superimposition techniques (Bookstein, 1991). Anthropology was one of the first fields applying such new toolkits (Slice, 2005), which were soon used to investigate integration of the human skull in ontogeny and evolution (e.g., Bookstein et al., 2003; Bastir and Rosas, 2005; Bastir et al., 2006).

According to these perspectives in structural morphology, one of the main goals is to investigate the degree and patterns of integration within a given anatomical system, or alternatively, the separation of modules characterized by highly correlated traits (Cheverud, 1982, 1996; Mitteroecker and Bookstein, 2007; Wagner et al., 2007). This approach provides essential biological information considering at least two different levels of analysis. In terms of phylogeny, the patterns of correlations among characters orientate and constrain evolution, facilitating some changes, or conversely, precluding some others. In terms of biology, the network of relationships among traits represents the scheme underlying the actual observed phenotypic variability (what we can call the essential “biological model” behind the morphotype). In morphometrics it can be useful to separate *variation* (the actual distribution of a phenotype) and *variability* (the intrinsic possibility to vary, changing the distribution of the variation; Wagner and Altenberg, 1996). Most of all, when considering the factors involved in intra-specific differences (Martin and Barbour, 1989), analysis of covariation structure is able to quantify *variation* and at the same time disclose the combination of characters channeling and constraining patterns of *variability*. That is, correlation patterns reveal those sets of rules based on structural and functional relationships among anatomical components, which are the ultimate product of the biological organization behind normal and pathological conditions. It is apparent that this same information is relevant in evolutionary biology as well as in biomedicine.

### FUNCTIONAL MORPHOLOGY AND EVOLUTIONARY NEUROANATOMY

The relationship between skull and brain is certainly a major topic within the field of functional craniology (Richtsmeier et al., 2006; Bruner, 2007). In fact, the system also includes the meninges and the vascular network, which act like functional and structural components within the morphogenetic processes (Moss and Young, 1960). Imbalances among these elements due to genetic or epigenetic factors are often associated with pathological and sub-pathological conditions, due to altered patterns of the ossification process (e.g., Morriss-Kay and Wilkie, 2005; Carter and Anslow, 2009; Richtsmeier and Flaherty, 2013). In terms of cranial integration, the facial and neural blocks are partially separated, but both interact with the cranial base (Bastir et al., 2010). The cranial base plays a major architectural role in primate ontogeny and evolution (Lieberman et al., 2000), and relevant biomechanical interactions bridging these districts are associated with the ethmo-maxillary complex (Enlow, 1990; McCarthy, 2001). In general, the cranial base is influenced by complex and multifactorial processes, including brain morphogenesis, posture, speech, thermoregulation, and so on. As a consequence, the three endocranial fossae are not strictly integrated according to specific morphological schemes, but rather are influenced by distinct and local factors (Bruner and Ripani, 2008). On the other hand, cranial vault morphogenesis has dynamics which are simpler and easier to investigate, and are mostly associated with brain pressure distribution during growth and development (Moss and Young, 1960).

During morphogenesis, imbalance among tissues may lead to non-pathological variants called “epigenetic traits” (Hauser and De Stefano, 1989; Manzi et al., 1996), and cranial morphogenesis is particularly sensitive to bone deposition and resorption associated with growth fields of osteoblasts and osteoclasts (Martínez-Maza et al., 2006). In this context, a lack of fine-tuned matching in size and shape changes between the brain and skull during ontogeny, can lead to the production or persistence of multiple centers of ossification like Wormian bones or persistent sutures, which are commonly described as non-pathological variants due to defects in the ossification process (*hypostotic traits*).

Neanderthals are an interesting case study in this sense (Bruner, 2014), as they retain plesiomophic patterns of relationships in their midsagittal brain morphology, in which the parietal areas are constrained between the frontal and occipital regions (Bruner, 2004). A primitive allometric pattern such as this, which is associated with their large brain volume, involves a spatial flattening of the parietal outline. At the same time, with regard to the neurocranial counterpart, Neanderthals frequently display supernumerary ossicles, which have been interpreted as “morphological instability” (Sergi, 1934, 1948). Such frequency has been measured as “*hypostotic scores*,” and interpreted as a structural result of ontogenetic stress (Manzi et al., 2000). Therefore, we must evaluate the possibility that in Neanderthals, a derived brain size associated with plesiomorphic patterns of cerebral growth and development, may have involved certain structural constraints, and potential problems associated with brain/skull morphogenetic relationships.

Relationships among brain and braincase are relevant to investigate evolutionary changes, and at the same time represent a fundamental topic in medicine. In general, any geometrical correlations among brain and skull landmarks are necessary to understand paleoneurological changes, as well as for planning surgical operations (Ribas et al., 2006; Richtsmeier and Deleon, 2009). Knowledge on the spatial relationships among cranial and cerebral landmarks can provide relevant information that can contribute to paleoneurological analysis and surgical practice.

**FIGURE 1:**
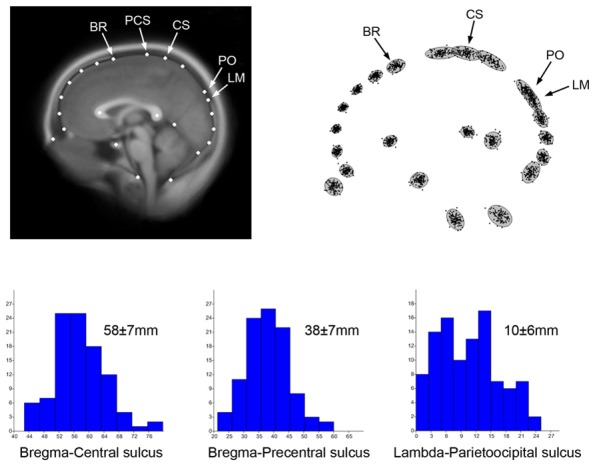
**Two-dimensional coordinates from 100 adult human MRI midsagittal images have been sampled to localize elements of the brain and skull, including the position of endobregma (BR), endolambda (LM), central and precentral sulcus (CS, PCS), and the parieto-occipital sulcus (PO).** Such configuration allows localization of the boundaries between frontal, parietal, and occipital bones and lobes. The average MR image (top left) has been obtained after Procrustes superimposition to minimize shape differences (Bookstein, 1991) using tpsSuper 1.14 (Rohlf, 2004). The superimposed coordinates (top right) show the distribution of the shape residuals after normalization of the form differences. The histograms show the distribution of the absolute distances between brain and cranial landmarks, with average and standard deviation. The distance bregma-central sulcus represents the distance between frontal bone and frontal lobe. The distance bregma-precental sulcus represents the part of prefrontal lobe covered by parietal bone. The distance lambda-parietooccipital sulcus represents the distance between occipital lobes and occipital bone. These spatial relationships between brain and braincase are of special interest for both paleoneurology and surgery.

In **Figure 1** 100 adult midsagittal brain sections have been sampled from the OASIS database (Marcus et al., 2007) and 2D landmarks have been selected from cranial and cerebral anatomical references. Magnetic resonance is in fact recognized for its ability to visualize brain, but it can also be used to localize sutures, because of their connective composition (Cotton et al., 2005). Superimposition techniques like Procrustes registration are aimed at minimizing the geometric differences among individuals through a normalization procedure, to investigate shape, degree of variation and patterns of variability through a multivariate analysis of the residual spatial dissimilarities (Bookstein, 1991). Such an approach can be used to characterize and quantify the covariation and correlation between cranial and cerebral elements. The scatterplot of the superimposed coordinates shows the distribution of landmarks, and indicates overlap between lambda and the parieto-occipital sulcus. Therefore, although the boundary between the parietal and occipital lobes generally lies behind the parietal bone, it may reach the occipital squama in some specimens. In fact, lambda lies beyond the parieto-occipital sulcus in 10% of the individuals in this sample. The raw Euclidean distance among major cranial and cerebral points can also be computed, and its distribution can be analyzed in a sample population. It is worth noting that parietal bones reach a stable morphology before the frontal bones during ontogeny, which is likely due to the later influence of facial morphogenesis on the frontal areas (Young, 1957). Hence, changes are fixed on the posterior vault first, and on the anterior vault later on.

This information on the patterns of spatial relationships between the brain and braincase, which is essential in surgical practice, is also necessary to make proper inferences about brain anatomy in paleoneurology, when the reconstruction of cerebral morphology is strongly based on cranial and endocranial form.

### FUNCTIONAL MODELING

**FIGURE 2:**
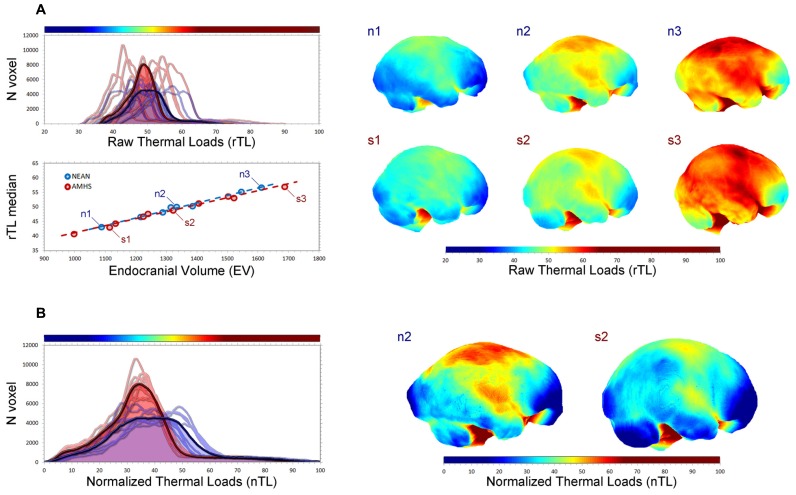
**Endocranial geometry can be useful to simulate metabolic heat production and dissipation in fossil species (Bruner et al., 2011a, 2012).** In this figure endocranial heat dissipation patterns in 10 living humans (five males and five females) and eight Neanderthals (Abri Suard 51, Gibraltar 1, Guattari 1, La Chapelle aux Saints, Saccopastore 1, Spy 1, Spy 2, Tabun) have been compared after numerical simulation, by applying the heat equation to their endocasts. Three modern humans (s) and three Neanderthals (n) of similar size (1, 2, 3: small, medium, large) have been compared. In **(A)** the distribution of the thermal values for each surface voxel (top left) is shown for modern humans (red) and Neanderthals (blue), for each individual and for their average curve (bold lines). The correlation between endocranial volume and median thermal values (bottom left) shows that size is the major determinant of the overall figure. Mapping the values (right) allows the visualization of local changes associated with size differences. In **(B)** heat values are normalized according to their range distribution, from 0 to 100. Despite the similar size, modern humans and Neanderthals display different curves, suggesting local differences in warming/cooling patterns of the endocranial surface as a function of their geometry. The normalized maps display such differences for the medium-size specimens.

Beyond morphology, numerical modeling can also provide functional information whenever some specific biological factor is associated with the spatial organization of neural tissues. Correlation between form and function can be used to investigate physiological processes from morphological evidences. In this sense, brain metabolism and thermoregulation are good examples of biological functions that may be modeled based on simple mechanistic and geometric principles (Nelson and Nunneley, 1998; Van Leeuwen et al., 2000; Sukstanskii and Yablonskiy, 2006; Zhu et al., 2006; Bruner et al., 2011a, 2012). Brains are well known for being among the most energy-demanding organs of the body, burning large quantities of glucose for the development and maintenance of their structural and functional integrity (Mink et al., 1981; Aiello and Wheeler, 1995; Leonard et al., 2007). Taking this into account, and considering that most energy released from the oxidation of glucose is lost as heat, metabolic heat production becomes another basic feature of all neural systems. Moreover, cerebral tissues are very sensitive to temperature changes, in that a slight increase of about 0.5–1.0°C may induce structural and functional changes at the cellular, histological and systemic levels, while severe and irreversible neural damage, coma, or even death of the individual may happen under hyperthermic conditions with brain temperatures above 40°C (Kiyatkin, 2010; Bertolizio et al., 2011; Rango et al., 2012). Therefore, it seems straightforward that brain thermoregulation processes are relevant at an evolutionary level, as brain temperature homeostasis may impose pervasive selective pressures and constraints on the evolution of species, and particularly on species-specific encephalization processes (Falk, 1990; Hofman, 2001, 2012; Caputa, 2004; Bruner et al., 2011a, 2012; Manger et al., 2013). Additionally, thermal management of neural mass is relevant in a biomedical context, as higher cerebral temperatures have been found in patients suffering from traumatic brain injuries or cerebral ischemia and stroke, as well as in other neurological disorders like schizophrenia, Parkinson’s disease, epilepsy, and multiple sclerosis (Kiyatkin, 2010; Bertolizio et al., 2011; Rango et al., 2012). In this context, brain temperature depends on the interplay between metabolic heat production and heat dissipation processes, with both factors being somewhat influenced by the gross geometry of the brain. While heat production and active removal of heat by cerebral blood flow are correlated with overall cerebral dimensions (Karbowski, 2007, 2009; Herculano-Houzel, 2011), passive heat diffusion (i.e., conduction) within the brain mostly depends on cerebral shape (Bruner et al., 2011a, 2012). Consequently, despite the fact that brain size is the main factor involved in overall thermal balance, local morphological changes may influence local cortical values associated with tissue warming/cooling. In this case, a comparison between modern humans and Neanderthals can be of interest for thermal biologists, taking into consideration that these two human taxa share the same cranial capacity, but different brain morphology (**Figure 2**).

Beyond form and function, even when considering specific traits paleontologists and surgeons are often interested in common anatomical information. Craniosynostoses and suture dynamics are other essential topics in both fields, being associated with morphogenetic factors involved in pathology and phylogeny (Di Ieva et al., 2013). Endocranial vascularization is another field in which paleoanthropologists and medical doctors share biological interests, taking into consideration the vascular differences described among extant and extinct hominids, and the importance of the same characters in a medical context (Bruner and Sherkat, 2008).

In the last two decades, growing attention has been placed on the relationship between evolutionary constraints and pathology, which has contributed to the development of perspectives in medicine that are based on an evolutionary foundation (Williams and Nesse, 1991). Taking into consideration the shared interests between biomedical fields and paleoneurology, here we introduce two case-studies in which paleontological information can add complementary approaches for understanding the processes behind the origin and etiology of two widespread pathologies like myopia and Alzheimer’s disease (AD), which have been hypothesized to be associated with changes at the frontal and parietal areas, respectively.

## BRAIN EVOLUTION AND MYOPIA

### FRONTAL LOBES

Increasingly, the importance of understanding the interchange between evolutionary biology and physiological function is being realized, and particularly in the context of evolutionary trade-offs associated with reorganization and differential development of the various regions of the brain during hominid evolution. Because organisms are not a collection of independent traits, but rather integrated entities (Moss and Young, 1960; Gould and Lewontin, 1979; Enlow and Hans, 1996), it is important to consider variation among spatially proximate features of the skull, and how long-term evolutionary trends may impact their functional capacities in a modern context.

An increase in absolute and relative brain size is arguably the main hallmark of the human evolutionary lineages, and is generally associated with evidence of increased cultural and behavioral complexity (see Sherwood et al., 2008). These changes in cerebral dimensions are integrated with modification to underlying basicranial and facial structures, and have been associated with an anteroposteriorly shorter face (Bookstein et al., 2003; Bastir and Rosas, 2009; Bastir et al., 2010). The extent to which the entire skull has rotated and the face and orbits have become tucked up under the brain is a unique derived feature of anatomically modern humans (Enlow, 1990; Lieberman et al., 2002; Bastir et al., 2008; Cobb, 2008).

Relative to body size the human brain is very large, with a majority of this increase beginning during the Middle Pleistocene (Ruff et al., 1997; Rightmire, 2004). Since this time there has been a nearly twofold increase in cranial size, however, enlargement of the various structures that make up the brain housed within it have not increased isometrically (Rilling, 2006). For example, volume of the human temporal lobes is larger than expected according to the proportions exhibited among living apes (Rilling and Seligman, 2002).

There is a longstanding debate about relative size of the frontal lobes. Because of their cognitive relevance, it is often assumed that the volume of these areas should have increased during human evolution, although proper evidence is lacking. Preliminary data suggested that the volume of the human frontal lobes is in line with what we would expect for a primate of the same brain size (Semendeferi et al., 1997; Semendeferi and Damasio, 2000). Despite this volumetric result, we may posit that the human frontal lobes at least exhibit an increase in their degree of connectivity when compared with living apes (Schoenemann et al., 2005; Rilling, 2006). However, the precise boundaries of these conventional areas are difficult to assess, and the comparative samples are relatively small. As a consequence, most comparisons do not reach the common statistical thresholds of significance (Barton and Venditti, 2013). Two criticisms can be offered with regard to the lack of evidence. First, minor differences cannot reach significance in terms of statistics, but they may be relevant in terms of biology. Second, even if modern humans have frontal lobe volumes consistent with what would be expected for a primate of that cerebral size, the absolute volume is three times the value of living apes, and such a patent increase in brain mass can well have a major effect on brain functions (Alba, 2010).

Even if we still lack definitive results concerning volumetric changes in the frontal lobes, we do possess some minor evidence of paleoneurological changes in terms of their form and proportions. As far as this can be observed in endocranial casts, the modern sulcal and gyral patterns at the frontal lobes can be observed in every human species, dating back 2 million years (Tobias, 1987; Holloway, 1995). A marked increase in their general proportions can be seen later in Neanderthals and modern humans, which show relatively wider prefrontal areas at the Broca’s cap when compared with other human species (Bruner and Holloway, 2010). It is likely that the lateral redistribution of this cortical mass may be related to constraints between brain and cranial structures, and in particular, constraints imposed by the underlying facial block (Enlow, 1990). Neanderthals, with a larger facial block, display even wider frontal areas when compared with modern humans, although differences are not significant, which may be due to the limited sample size.

**FIGURE 3:**
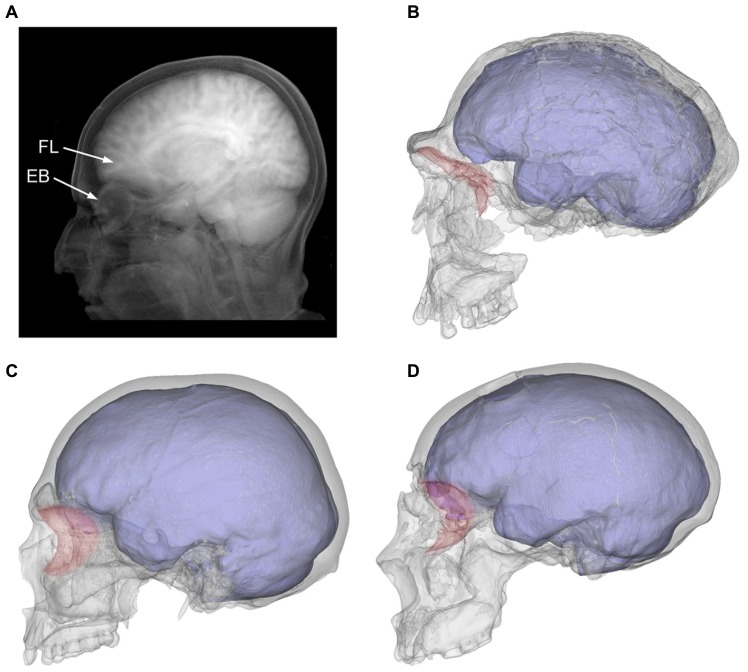
**The tomographic projection shows the intimate proximity between frontal lobes (FL) and the eyeball (EB) in a modern human individual A).** The orbits (in red) were separated from the anterior cranial fossa in archaic humans like *Homo ergaster* (**B**; KNM-ER 3733), but moved under the prefrontal areas in modern humans (**C**; modern European) and Neanderthals (**D**; Saccopastore 1). It can also be seen how the expanded temporal lobes become more closely associated with the posterior orbit in modern humans when compared to these other taxa.

Hence, in evolutionary terms the anterior fossa is free to change laterally, because in the human genus the orbits are displaced anteriorly, and the temporal muscle is extremely reduced because of marked muzzle reduction (Bruner, 2004). By contrast, vertical increase of the frontal areas is constrained in these two taxa by a specific biomechanical limitation, which is that in modern humans and Neanderthals the frontal lobes lie directly on top of the orbital roof (Bruner and Manzi, 2008). Taking into account that such close contact is not observed in less encephalized hominids (Bookstein et al., 1999; Bruner and Manzi, 2005), we must assume that this form, involving greater interaction between these two anatomical components, has evolved in both lineages independently beginning about 100–150 ky ago (**Figure 3**).

### VISUAL IMPAIRMENT AND FUNCTIONAL CRANIOLOGY

Encephalization within the hominid lineage has resulted in a number of modifications to the cranial vault, cranial base, and face, which together comprise the major structural components of the skull. The large brain of modern humans, and its influence on craniofacial development throughout ontogeny, has been hypothesized to be associated with secondary problems such as choking, dental crowding, impacted dentition, as well as a reduction in olfactory and masticatory function (Ross, 1995; Lieberman et al., 2000; Ravosa et al., 2000). A more anterior position and lateral development of the prefrontal cortex above the eyes, expansion of the temporal lobes posterior to them, and reduced facial prognathism below, may also impact vision in a functional sense. These morphological trends could have a deleterious effect on visual acuity, as they act to constrain orbital and ocular development in modern humans, considering that changes in one trait can negatively impact other neighboring elements (Moss and Young, 1960; Enlow and Hans, 1996; Bruner, 2007; Martínez-Abadías et al., 2012).

Constraints upon the orbits and soft tissues of the eye, associated with expansion and anterior relocation of the frontal and temporal lobes, could have been more severe in modern humans and Neanderthals than in small-brained hominids, as a result of the unique spatial relationship among these anatomical traits in these groups. In an ongoing study we are currently evaluating whether greater midfacial prognathism and larger orbits in Neanderthals (Schultz, 1940; Masters, 2009; Pearce et al., 2013) may have partially limited such spatial conflicts. Independent of the degree to which this extinct taxon may have experienced constraints, modern humans are characterized by enlargement and forward movement of the anterior and middle cranial fossae, forward projection of the greater sphenoid wings, and rotation of the posterior maxillary plane (McCarthy and Lieberman, 2001; Lieberman et al., 2002; Bastir et al., 2008; Cobb, 2008), as well as a reduction in anterior projection of the orbital margins and internal orbital depth (Masters, 2009).

These evolutionary changes, in addition to an observed decrease in orbital volume since the Neolithic in China (Brown and Maeda, 2004; Wu et al., 2007), are important trends to consider in investigating the increased frequency and severity of certain forms of reduced visual acuity like myopia (nearsightedness) and astigmatism. Taken together, these morphological trends would be expected to result in a more forward projecting (exophthalmic/proptotic) eye, which would become anterior to the concave segment of the orbital roof and floor in which it is meant to rest. Because the inferior and superior orbital margins curve toward each other, the eye and contiguous muscles and fat may become compressed and distorted as they move into a more anterior position.

A strong negative correlation between proptosis and spherical equivalent refractive error (meaning vision becomes worse as the eye projects outward from the orbit), has led some researchers to suggest that degree of refractive error be considered in studies of exophthalmia, as it was presumed that increased axial length of the eye in myopes causes it to protrude out from the orbit (Migliori and Gladstone, 1984; Quant and Woo, 1992). However, it is likely that decreased orbital depth, in association with increased frontal and temporal lobe development, forces the eye into a more proptotic position beyond the concave aspect of the orbital margins. This anterior relocation of the globe may result in increased curvature of the cornea and axial elongation of the eye, as a result of superoinferior pressure being applied to the eye and extraocular tissues as they shift forward toward the smaller part of the orbital opening during ontogeny. This alternative model suggests that increased axial length of the myopic eye is not a contributing factor to exophthalmia, but rather a result of its anterior placement, protrusion beyond, and compression against the narrowing rim of the orbit.

This eye form, which is hypothesized to result from orbital constriction associated with the unique trajectory of hominid cerebral and craniofacial evolution, is perhaps not coincidentally the most common eye form associated with the development of astigmatism and juvenile-onset myopia in humans. Myopic refractive errors such as these are the most common disorders of the eye in humans, and the etiology of these conditions is still unknown. Astigmatism is associated with irregular curvature of the cornea, and myopia with an overly large, axially elongated eye, increased vitreous depth, and increased focusing power of the cornea, which cause an image to be erroneously focused in front of the retina (Curtin, 1985; Working Group on Myopia Prevalence and Progression, 1989; Lam et al., 1999; Stone and Filtcroft, 2004; Dirani et al., 2006). Among these and other factors that can influence myopic progression, axial length of the eye has been found to be the biggest contributor to the condition, and particularly among individuals over 12 years of age and of East-Asian ancestry (Ip et al., 2007), where myopia is so common that it affects 80–90% of individuals in some East-Asian populations (Goldschmidt et al., 2001; Lin et al., 2004; Park and Congdon, 2004). It is also found to occur earlier in life and at a higher frequency among Chinese schoolchildren compared to individuals of African or European descent (Lam et al., 1999; Ip et al., 2007, 2008).

In addition to being axially elongated, the eye of myopes is also ubiquitously larger (Zadnik et al., 1994; Ip et al., 2007; Lam et al., 2008), though it is still unknown why the eye exhibits these characteristics in those with nearsightedness. However, a recent analysis of published data on eyeball volume, orbital volume, and refractive error in Chinese adults has indicated that relative size of the eye within the orbit may be an important predictor of myopia (Masters, 2012). Here it was shown that individuals with large eyes in small orbits have a higher rate of myopia and a greater degree of refractive error, while those with smaller eyes in relatively large orbits retain much more acute vision. This indicates that it is not simply absolute size of the eye itself, but rather its relative volume within the hard tissue confines of the orbit that influence the development of this condition.

Interestingly, reduced visual acuity is also common among numerous dog breeds such as the Toy Poodle, Miniature Schnauzer, Pug, Rottweiler, Collie, and Labrador retriever (Murphy et al., 1992; Kubai et al., 2008; Williams et al., 2011). Varieties with a higher frequency and greater severity of refractive error also tend to be those that have undergone greater human-imposed selective forces applied to their craniofacial architecture, because of functional and/or esthetic reasons. The majorities are generally characterized by craniofacial paedomorphism resulting from artificial selection for retention of juvenilized traits, and possess a more frontated and globular neurocranium, shorter face, and proptotic eyes. For example, an analysis of naturally occurring myopia among three separate dog breeds showed that the condition was far more prevalent in Toy Poodles (63.9%), compared with less paedomorphic breeds like the English Springer Spaniel (36.4%), and Collie (35.7%) (Williams et al., 2011).

In many studies of dogs with naturally occurring myopia, the condition has been found to progress in much the same way as it does in humans. This has been demonstrated most clearly in the Labrador retriever, where a significant genetic component exists (Black et al., 2008), and the greatest contributors to refractive error include a thinning lens and increased vitreous chamber depth (Mutti et al., 1999). Because of the necessity of finding food, recognizing others, and being aware of dangers and benefits in an environment, it is only recently that such visual detriments could begin to occur in dogs and humans (Cordain et al., 2002). Though given the high level of variation in both craniofacial form and myopia prevalence rates among different breeds of dog, we must wonder if the greater frequency and severity of refractive errors in those with shorter faces, more frontated crania, and relatively large proptotic eyes may parallel the human condition, given the ubiquity of these trends throughout hominid evolution.

Although the eyeball lies predominantly within the orbit, it does not directly influence orbital size in humans (Schultz, 1940; Chau et al., 2004), but rather eye growth keeps pace with growth of the brain (Salzmann, 1912; Todd et al., 1940; Weale, 1982), and both are thought to be the product of pleiotropic gene control (Miller, 1992; Mak et al., 2006). By contrast, the orbit grows with the rest of the cranium (Waitzman et al., 1992), and has been shown to vary in association with overall body size to the extent that body mass and area of the orbital opening are correlated at *r* = 0.987 (Kappelman, 1996). If growth of the eye and brain are a product of pleiotropy, prolonged brain growth during human evolution and in association with learning throughout life, would also act to increase size of the eyeball. At the same time, this concurrent cerebral development would limit available space for the growing eye and extraocular tissues within the orbit, as a result of bilateral and anterior development of the frontal and temporal lobes above and behind. Because dimensions of the orbital margins and body mass are highly correlated (Kappelman, 1996), and a negative allometric relationship exists between the eye and orbit with respect to body size (Schultz, 1940), the well-documented reduction in overall human size and robusticity that began 12,000 years ago (Carlson, 1976; Carlson and Van Gerven, 1977; Smith et al., 1985, 1986; Henneberg, 1988; Henneberg and Steyn, 1993; Lahr and Wright, 1996) would act to increase the percentage of the orbit occupied by the eye, and bring humans as a whole closer to the point at which these tissues vie for space. Additionally, the observed decrease in orbital volume in East Asia (Brown and Maeda, 2004) could exacerbate an existing trend toward increased relative size of the eye within the orbit, and potentially help explain the unusually high frequency of myopia in this geographic region.

Humans have experienced a unique morphological history among mammals, in which millions of years of cerebral expansion and reduced facial prognathism have brought the eyes and orbits to rest directly beneath the frontal cortex. Considering these prominent evolutionary trends, certain forms of reduced visual acuity like astigmatism and myopia may relate to competition among neighboring hard and soft tissues of the skull, and specifically cerebral and craniofacial constraints upon ocular tissues within the orbit of modern humans. Despite over 100 years of research it is still unknown what causes astigmatism and myopia, and why it is consistently found to correlate with factors such as sex, ancestry, age, intelligence, and socioeconomic status. The longstanding idea that near work is to blame for myopia, which had been advocated for over 400 years, is also no longer supported, as it has yet to be shown how convergence and eye strain associated with more reading and near vision work can permanently alter the shape of the eye, and no other environmental risk factors that alter ocular growth have been identified (Saw et al., 2006; Mutti and Zadnik, 2009; Mutti, 2011). By contrast, a broader approach rooted in evolutionary anatomy, modern human variation, ophthalmology, paleoneurology, and biomedicine may add to a better comprehension of the anatomical relationship among hard and soft tissue components of the visual, craniofacial, and cerebral systems, and how evolutionary benefits resulting from change in one feature may negatively impact neighboring traits during evolutionary and ontological morphogenesis.

## PARIETAL LOBES AND ALZHEIMER’S DISEASE

### THE EVOLUTION OF THE PARIETAL AREAS

Brain geometry has always represented a major issue in evolutionary studies, in terms of form, shape, and size variations (Hofman, 1989, 2012). On a large scale, dimensions, proportions, and spatial organization may have been relevant factors in functional changes associated with the overall organization of the brain system. On a smaller scale, local structures can be influenced by geometrical and physical properties at even the cellular level (Van Essen, 1997; Hilgetag and Barbas, 2005; Toro and Burnod, 2005). Because of such fine biomechanical balance associated with morphogenesis and histological properties, minor changes in the physical composition of the connective, osseous, or neural elements of the brain, can exert direct changes in the spatial association of endocranial components.

**FIGURE 4:**
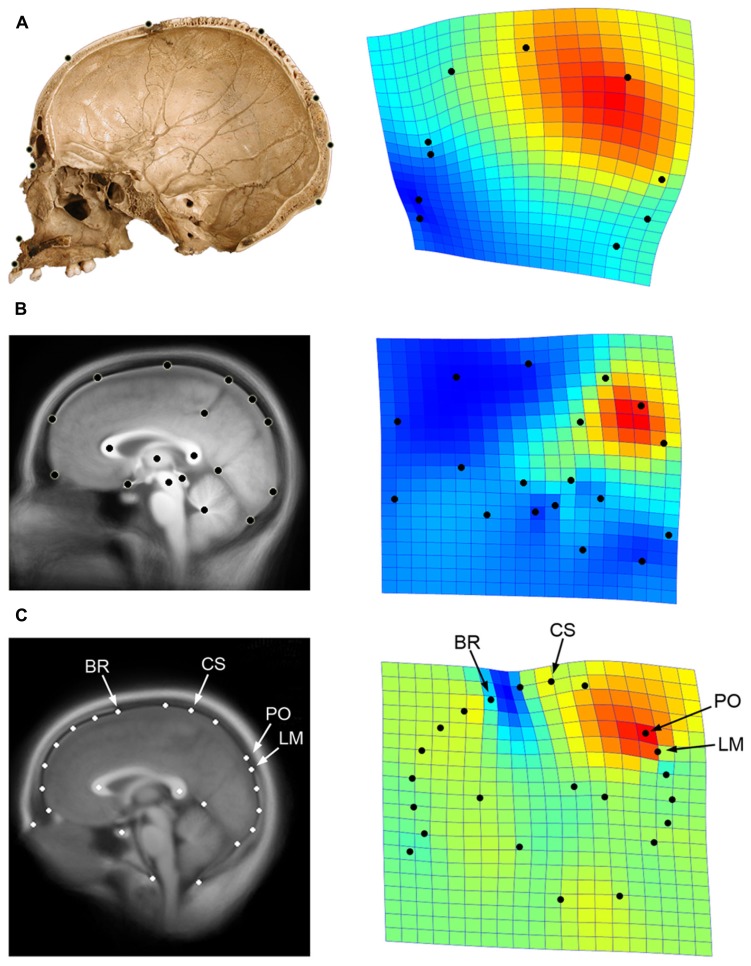
**Parietal lobe dilation is responsible for the major differences in neurocranial morphology between modern and non-modern humans (A; data after Bruner et al., 2004).** A similar pattern represents the main axis of variation within adult modern humans, particularly evidenced by the proportions of the precuneus in generating such differences (**B**; data after Bruner et al., 2014). If we consider both cranial and brain landmarks (**C**; see **Figure 1**), we can see that the bulging of the upper parietal areas associated with the first principal component approaches the central sulcus (CS) to bregma (BR), and shifts the parieto-occipital sulcus (PO) away from lambda (LM). The geometrical configuration used in each analysis is shown on the left, while the first principal components of variation after Procrustes superimposition is shown on the right through thin-plate spline deformation grids and deformation map (red: dilation; blue: reduction; data computed using PAST 2.17c; Hammer et al., 2001). Here it can be seen that the same pattern is associated with modern human origin and evolution, modern human variation, and skull/brain relationships.

Globularity of the braincase is one of the most ostensible cranial features of modern humans compared with extinct hominids (Lieberman et al., 2002; Bookstein et al., 2003). In terms of cranial profile, apart from a minor increase of the frontal curve (Bookstein et al., 1999; Bruner et al., 2013) the extent of globularity in *Homo sapiens* is due to bulging of the posterior vault surface, and is largely associated with a geometric dilation of the parietal bone (Bruner et al., 2004; **Figure 4A**). Such globular shape of the neurocranium is a discrete feature of our species, and we have no evidence of any extinct taxon with a gradual or intermediate phenotype. In Africa around 150–200 ky, we have specimens associated with the modern human lineage that display modern parietal bossing, while others lack such features (Bruner and Pearson, 2013). Therefore, we must suppose that modern globular morphology began to evolve at that time, among geographical variants of the late Middle Pleistocene in Africa.

Beyond the geometry of the neurocranial bones, a quantitative analysis of endocranial form and shape also provides evidence that modern humans are characterized by a species-specific upper bulging of the parietal areas (Bruner et al., 2003, 2011b; Bruner, 2004), and results are the same if we consider endocranial (bone) or cortical (brain) anatomical references. Recent paleoneurological inferences, based on correlations between cranial and cerebral morphology, suggest that Neanderthals had relatively larger occipital lobes when compared with modern humans (Pearce et al., 2013). This hypothesis fits with the larger relative parietal proportions in the latter group, taking into consideration the similar brain volume in these two taxa. Such spatial changes in parietal geometry are associated with an early postnatal stage in our species (Neubauer et al., 2009), which is absent in chimpanzees (Neubauer et al., 2010) and Neanderthals (Gunz et al., 2010).

As a matter of fact, since the earliest studies in paleoneurology, the parietal lobes were recognized to be surprisingly variable among hominoids, and also show marked differences among human species when analyzed with traditional or geometrical approaches (Weidenreich, 1936; Holloway, 1981). It is worth noting that although lower parietal areas like the supramarginal and angular gyrus have a relevant cognitive role in our species (like in speech understanding), to date, the paleoneurological record has evidenced no patent differences in their raw morphology between modern humans and other large-brained hominids like Neanderthals. Instead, morphological changes in the parietal regions among hominoids are probably associated with deep and upper parietal volumes, like the precuneus or the intraparietal sulcus, which are largely involved in visuo-spatial integration (Bruner, 2010; Bruner et al., 2014).

Interestingly, if we analyze the midsagittal profile of the brain in a sample of adult modern humans, we can see that fronto-parietal bulging is also the major source of intra-specific variability (Bruner et al., 2010), suggesting the probable role during human evolution of lines of least resistance, in which intra-specific variability facilitates and orientates phylogenetic changes (Schluter, 1996).

A recent geometric analysis has shown that relative dimensions of the precuneus are largely responsible for these major patterns of variation (Bruner et al., 2014; **Figure 4B**). Despite the difficulties in defining the exact boundaries of these cortical areas, the results clearly showed that the upper region of the precuneal area is strictly involved in generating the largest differences within the sample. Sexual differences and size-related effects are, in this case, absent or negligible.

Because of spatial packing of the brain onto the endocranial base (Ross et al., 2004), neurocranial globularization could be hypothesized to be a secondary consequence of cranial base flexion. Nonetheless, current evidence does not support this possibility. Neanderthals and modern humans share the same brain size, and possess only a minor difference in cranial base flexion, which can hardly explain the large differences in brain shape. Most importantly, the relationship between encephalization and the cranial base angle is not clear (McCarthy, 2001), suggesting that the factors involved are not patently correlated. We must also remark that shape changes associated with a simple bending of the cranial base are not necessarily associated with an absolute expansion of the cortical areas involved, which is the case in modern human parietal morphology. Finally, in adult modern human variation the expansion of the precuneus is not associated with flexion of the subcortical and basal geometry. Therefore, despite the fact that cranial base flexion may have played a role in general spatial brain organization, its possible role in parietal bulging among anatomically modern humans is not necessarily apparent.

We can wonder whether such changes in brain form may influence or be influenced by changes in spatial organization of the neurocranial bones. Using the same data presented previously (see **Figure 1**), and by including cranial landmarks like bregma and lambda, we can compute a Principal Component Analysis on the shape residuals to evaluate the patterns of spatial relationships among the neurocranial bones and the frontal, parietal, and occipital lobes (**Figure 4C**). By using cortical, subcortical, and cranial landmarks after Procrustes superimposition, we can see that the first component of variation is again associated with upper parietal bulging. Interestingly, such geometrical dilation of the precuneal region has an effect on the spatial relationships among the skull and brain. For example, as the parietal area increases, the somatosensory area approaches the frontal bone, and the perpendicular (parieto-occipital) sulcus shifts away from the occipital bone. By contrast, in brains with flatter parietal regions, bregma shifts away from the central area, and the perpendicular sulcus approaches lambda. Therefore, instead of a homogeneous response between bones and lobes, we have a change in the relative position of these anatomical elements along this major axis of covariance. With regard to this observed pattern, the precentral sulcus never reaches the frontal bone, but the parietal lobe can reach the occipital bone (the perpendicular sulcus can encroach upon the position of lambda). As mentioned, this information is essential to both paleoneurologists and surgeons, in that it provides a better understanding about relationships among the skull and brain associated with parietal expansion.

Taking into consideration these results, from the extant and extinct patterns of morphological neurocranial variation, it is apparent that the same source of geometric variability (dilation of the deep parietal areas) plays a major role in evolutionary changes, intra-specific differences, and brain/skull organization.

Apart from the morphological evidence, more information has recently been added to the body of research involving the deep parietal areas, which were once regarded as just general “associative areas.” In terms of cytoarchitecture, humans have specific cellular areas in the intra-parietal sulcus when compared with non-human primates (Orban et al., 2006). The parietal areas also represent the main node of integration between structural and functional brain networks (Hagmann et al., 2008), and because of their complex integration with the frontal areas, are particularly relevant in current theories on intelligence (Jung and Haier, 2007). Fronto-parietal integration is also a basic component of imitation, which is a cognitive ability unique to our species (Hecht et al., 2013).

The intermediate geometrical position of the parietal elements makes them sensitive to most of the changes affecting the rest of the brain, including physical constraints associated with morphogenesis and evolution (Bruner, 2004). Because of their spatial position, the deep parietal areas represent a structural and functional bridge between all other brain regions, and are therefore more sensitive to any changes or constraints exerted in other specific areas. In terms of structure, these areas are constrained between the frontal and occipital extremes, and their morphology must adjust according to the general spatial arrangement of the endocranial cavity, for which variations are further channeled by connective tensors like the falx cerebri (Moss and Young, 1960). In terms of functions, the deep parietal areas display major connections with the frontal lobes, an intimate continuity with the occipital lobes, a tight relationship with the subcortical elements (Cavanna and Trimble, 2006; Zhang and Li, 2012), and evolutionary changes among them must necessarily be integrated in such systems. Therefore, we cannot discount that part of the evidence associated with the deep parietal areas is a direct or indirect consequence of their crucial spatial position, beyond the specific functions of the parietal lobes. Nonetheless, at the same time we cannot discard that evolutionary changes in these cortical components may have played a principal role in cognitive or physiological functions. In terms of cognition, these areas are largely responsible for visuo-spatial integration, including coordination of the inner and outer environment through the eye-hand “ports,” and generation of an inner virtual space to perform mental experiments and simulations (see Bruner, 2010, 2012 for a review). This is particularly relevant in cognitive sciences, considering current theories on the extended mind, in which the integration among brain, body, and environment are essential to generate our behavioral capacities (Iriki and Sakura, 2008; Malafouris, 2009; Iriki and Taoka, 2012).

### BRAIN EVOLUTION AND NEURODEGENERATION

A patent change in a specific brain area requires relevant adjustments in the vascular system and in the metabolic balance within the brain. Interestingly, the middle meningeal vessels display a complex and reticulated morphology in only modern humans, but not other hominids, and especially on the parietal surface (see Bruner and Sherkat, 2008). Although such differences are macroscopic, we still ignore the functional and evolutionary meaning of this specific change (Falk, 1993; Bruner et al., 2011a). Nonetheless, considering that the vascular systems share common morphogenetic factors, the complexity of the middle meningeal artery in our species may reasonably suggest that, in general, modern humans display a more complex endocranial vascular system than extinct human species. The neurocranial vascular networks (cerebral, meningeal, diploic, and pericranial) are theorized to be particularly relevant in brain thermoregulation, taking into account that this organ requires a large amount of energy, is sensitive to temperature changes, and ostensibly has no other specific mechanism for cooling/warming the cortical mass. Therefore, taking into account size increase due to encephalization, and shape changes associated with globularity, we may wonder whether increased complexity of the vascular network in modern humans may be related to changes in thermal adaptations and metabolic responses.

The precuneus is a key component of the default mode network; namely the system involved in the baseline resting state of the brain, and is often suspended when the brain is engaged in specific tasks (Raichle et al., 2001). Interestingly, it also shows exceptional levels of energy management (Cavanna and Trimble, 2006; Margulies et al., 2009; Sotero and Iturria-Medina, 2011; Zhang and Li, 2012). As previously mentioned, for the deep parietal areas we must consider that the precuneus, approaching the core of the brain in terms of localization, can be susceptible to high thermal loads because of its spatial position (Bruner et al., 2012). A sphere has a lower ratio between surface (dissipating heat) and volume (producing heat), compared with a more ellipsoid geometric shape, and the deeper areas are more likely to accumulate heat by virtue of their location. The precuneus is a cortical element that is more sensitive to such constraints, and we must assume that in this sense brain shape changes associated with neurocranial globularity can affect this particular area to a greater extent.

Therefore, it appears that the same geometric changes that contribute most to modern human brain shape, intra-specific brain differences, and skull-brain architecture, are also those that are most associated with vascular variations and which are particularly sensitive to thermal management.

Alzheimer’s disease is a widespread neurodegenerative disorder, with devastating consequences for mental and cognitive capacities, health care management, and social factors (see Cummings, 2004; Dubois et al., 2010; Jacobs et al., 2012). It is generally characterized by accumulation of neuritic plaques composed of extracellular deposits of amyloid peptide, which leads to neuronal loss, cortical atrophy, and cognitive impairment. Even though plaques are considered to be a crucial causal factor of the disease, the amount of plaque does not correlate with cognitive impairments. Neurofibrillary tangles, most often in the medial temporal areas, are another main neuropathological feature of AD. In this case, although the quantity of tangles correlates with cognitive dysfunction, they are not strictly specific to this disease. Because of the importance of these structural damages in this neurodegenerative disease, and because atrophy in the early stages of the disease is most prominent in the medial temporal areas, AD studies have been strongly centerd on the temporal lobes. More recently however, it has been shown that in the earliest stages of the disease, metabolic impairments, neuroanatomical, and histological changes, can be found in the medial parietal cortex (Jacobs et al., 2012, 2013; Doré et al., 2013; Huang et al., 2013).

Clinically, AD pathology is explicitly found in only our species. Although extended lifespan in humans may involve problems of maintenance of the brain integrity (Sherwood et al., 2011), AD is not a general deterioration of the tissues, but a specific pathological impairment. Similar neurodegenerative processes can be seen in non-human primates in very rare cases, but never in the particular combination and expression which is typical (and so frequent) in humans. The specificity and high prevalence in our species may suggest an evolutionary linkage between its pathology and our brain biology. Consequently, we have a pathology that appears to be strictly associated with *H. sapiens*, beginning with metabolic and cellular impairments in those same brain areas which are associated with the origin of the modern human brain. Beyond any possible coincidence, we should seriously evaluate the possibility that the vulnerability of the parietal areas, and the sensitivity to the processes of neurodegeneration associated with AD, could be a drawback of the complexity of our brain anatomy (Bruner and Jacobs, 2013).

Metabolic or thermal loads, blood management, cellular turnover, or oxidative stress, may be some of the problems associated with complex and highly active tissues. Interestingly, in hibernating mammals the phosporilation of tau protein, which in AD accumulates in neurofibrillary tangles, is influenced by temperature, which is a delicate issue associated with heat production in the brain (Stieler et al., 2011). Apart from recognizing that changes localized in the parietal cortex may be directly responsible for constraints associated with initiation of the pathological processes, we must also remark that the delay in brain growth and development associated with our species can involve an additional stress in terms of energy balance, further increasing vulnerability to metabolic failures (Bufill et al., 2013). In any case, this risk of neurodegeneration at older ages does not affect genetic fitness, in that it influences a period which is generally beyond human reproductive stages. In terms of evolution, such a drawback can be interpreted as a case of antagonistic pleiotropy, in which cognitive advantages are paid with the costs of a powerful, expensive, and delicate anatomical system.

This evolutionary interpretation of AD can provide a different perspective with regard to the pathology of this disease, and most importantly, can orientate future investigations in a new direction. If sensitivity to AD is a consequence of our complex parietal biology, we should consider at least four main issues. First, we need more comparative studies investigating the deep parietal areas between humans and non-humans primates. Until now, difficulties in defining homologous references in parietal volumes among primates and other mammals have largely hampered quantitative and comparative analyses in this sense. Second, we should investigate whether these structures or networks, whenever localized, directly influence the etiology of AD. Third, we should evaluate structural (cells, vessels) vs. functional (metabolism, thermoregulation) factors that may possibly disrupt these areas. Fourth, we should consider how damage in the brain associated with this disease passes between the parietal and the temporal cortex. The fact that AD pathology topographically matches with disease-related alterations in hubs of the Default Mode Network may be indicative in this sense, taking into consideration that this system represents a principal and energy-expensive connective network that is constantly associated with intrinsic brain activity (Raichle, 2010).

## CONCLUDING REMARKS

The evolution of large brains represents a challenge in biology. In different periods throughout the phylogenetic history of the human genus we recognize a generalized encephalization process, associated with increasing behavioral complexity. Such an association, supported by theories correlating brain size and intelligence, has generated a debate which is, to date, still open (e.g., Balter, 2012). Though independent of any correlations with cognitive abilities or the primary selective processes behind such evolutionary changes (Alba, 2010; Manger et al., 2013), we know that big brains are expensive and difficult to handle in terms of ecology and anatomy. Increasing size by retaining plesiomorphic phenotypic patterns may lead to dead-ends and allometric constraints. In the case of Neanderthals, we have discussed how primitive structural patterns in the parietal areas may be associated with supernumerary ossification centers on the cranial counterparts, suggesting morphological instability due to imbalance in the integration of growth (size changes) and developmental (growth changes) patterns. At the same time, the evolution of derived features can introduce new factors which may not be well integrated in terms of structural and functional responses. A large brain in modern humans may have introduced functional and structural conflicts between the neural and facial districts, increased heat stress, and exposed the parietal areas to functional limitations. The integration of evolutionary and medical fields can provide robust theories and hypotheses to investigate the biological meaning of phylogenetic changes, and at the same time orient biomedical research according to a more comprehensive approach. Anthropologists and medical doctors have different questions, but they share numerous tools and objectives. The former used to have a more developed theoretical background, and the latter more complete functional information. As a matter of fact, we need to know the evolutionary process to understand a given pathology, and at the same time the evolutionary theories need a level of verification and quantitative evidence that can only be supplied by neontological studies and large samples. Multidisciplinary approaches are also necessary for the methodological aspects of research, to develop and enhance proper techniques that increase the analytical power of the current toolkits in digital anatomy and computed morphometrics. Of course, as always, caution is warranted. The risk of evolutionary medicine is an excessive adaptationism, devoted to explaining with finalistic and teleological approaches any observed variation. Many hypotheses in this field have large speculative components, due necessarily to the nature of the evolutionary evidence. In this sense, we should avoid the temptation to exaggerate with talkative proposals, reminding that science is based on *probability* and *interpretation*, rather than *possibility* and *explication*. With this limitation in mind, there is no doubt that evolutionary anthropology, as natural history of the humankind, can represent an interesting and informative key to evaluate failures and successes of our species in a biomedical context.

## Conflict of Interest Statement

The authors declare that the research was conducted in the absence of any commercial or financial relationships that could be construed as a potential conflict of interest.
